# Structural health monitoring (vibration) as a tool for identifying structural alterations of the lumbar spine: a twin control study

**DOI:** 10.1038/srep22974

**Published:** 2016-03-11

**Authors:** Gregory N. Kawchuk, Jan Hartvigsen, Tiffany Edgecombe, Narasimha Prasad, Jaap H. van Dieen

**Affiliations:** 1 Department of Physical Therapy, University of Alberta, Edmonton, Alberta, Canada; 2 University of Southern Denmark, Odense, Denmark; 3 Nordic Institute of Chiropractic and Clinical Biomechanics, Odense, Denmark; 4 Department of Mathematical and Statistical Sciences, University of Alberta, Edmonton, Alberta, Canada; 5 MOVE Research Institute Amsterdam, Department of Human Movement Sciences, Vrije Universiteit Amsterdam, Amsterdam, The Netherlands

## Abstract

Structural health monitoring (SHM) is an engineering technique used to identify mechanical abnormalities not readily apparent through other means. Recently, SHM has been adapted for use in biological systems, but its invasive nature limits its clinical application. As such, the purpose of this project was to determine if a non-invasive form of SHM could identify structural alterations in the spines of living human subjects. Lumbar spines of 10 twin pairs were visualized by magnetic resonance imaging then assessed by a blinded radiologist to determine whether twin pairs were structurally concordant or discordant. Vibration was then applied to each subject’s spine and the resulting response recorded from sensors overlying lumbar spinous processes. The peak frequency, area under the curve and the root mean square were computed from the frequency response function of each sensor. Statistical analysis demonstrated that in twins whose structural appearance was discordant, peak frequency was significantly different between twin pairs while in concordant twins, no outcomes were significantly different. From these results, we conclude that structural changes within the spine can alter its vibration response. As such, further investigation of SHM to identify spinal abnormalities in larger human populations is warranted.

The spine can be seen as an assembly of structures that act together to provide stability and mobility. While well-established techniques exist to evaluate the integrity and function of *man-made* mechanical assemblies, surprisingly few techniques exist to evaluate these same features in *biological* mechanical assemblies such as the spine^1^,^2^,^3^.

When assessing spine function, current best practices are to enquire about a person’s ability to perform functional activities (e.g. the number of stairs that can be climbed). While these self-reported measures can be reliable^4^, some studies suggest that recollection of physical function captures different phenomena compared to evaluation of function obtained by direct measurement or imaging^5^,^6^. While the ideal circumstance would be to acquire both self-reported and direct measures of spinal function, direct measures are currently problematic. Specifically, static visualization techniques such as magnetic resonance imaging (MRI) may not provide functional information^7^,^8^ while other assessment techniques can be difficult to interpret (e.g. electromyography)^9^,^10^, or are difficult to employ due to cost, access and invasiveness (e.g. fluoroscopy)^11^.

Given the above, there is a significant inability to quantify spinal function directly and when available, to interpret such information in a clinical context. Without increasing our ability to measure the mechanical function of the spine, existing deficiencies in the prevention, diagnosis and treatment of musculoskeletal conditions such as low back pain (LBP) are likely to continue and increase in cost. Presently, the one year prevalence of back pain is as high as 83%^12^ with total attributable costs in the US alone estimated to be between $84 and $625 billion dollars per annum^13^.

To fill this void, several investigators have identified established engineering techniques used in evaluating mechanical assemblies and adapted them for use in the spine. One of those techniques is structural health monitoring (SHM)^14^, an approach used routinely to evaluate structures as large as bridges and as small as electronic components. In SHM, vibration is passed through the object of interest and its response is then analyzed for deviations from the expected result. One underlying assumption of SHM is that the system being tested is linear in its response to vibration application. Specifically, linearity means that as the amplitude of the input vibration changes, the vibration response changes proportionally. While biological systems are typically non-linear in their response, biological systems may still be assessed with SHM techniques by obtaining a “linear equivalent” when the vibration amplitude at each frequency is remains constant, is small in magnitude, applied at the same site and yields acceptable signal coherence^15^.

Using this approach, invasive SHM techniques have been used in cadaveric studies^16^,^17^,^18^ and have been found to be a) highly reliable and b) able to identify the existence, location and magnitude of a variety of surgically-induced conditions (e.g. annular tears of the intervertebral disc). In addition, it has been shown that mechanical changes due to physiological or pathological changes (as opposed to surgically induced) can also been achieved with vibration testing^18^. Unfortunately, these approaches use invasive methods to connect sensors directly to the spine.

Given the above, our team developed a non-invasive form of SHM then applied it in a study design using identical twins with MRI determination of spine concordance as a gold standard. Twin designs have significant advantages compared to age/sex matching of unrelated subjects including a tendency to accentuate differences between twins, which makes findings of statistical indifference between twins very robust. With this design, the objective of this study was to determine if SHM could identify structural alterations in the lumbar spine of live human subjects as validated by MRI. The hypothesis of the investigation was that SHM data would not differ significantly in twin pairs with spinal concordance, but in twin pairs with spinal discordance, SHM data would differ significantly.

## Methods

### Subjects Recruitment and Twin Concordance

A list of potential participants from the Danish Twin Registry was generated based on back pain data collected in the 2002 omnibus survey^19^. With the aim of identifying monozygotic twin pairs discordant for back pain and structural damage to the lumbar spine, (i.e. one twin having had major trauma and/or surgery to the spine while the other had not), as well as identifying pairs concordant for back pain and pathology in the lumbar spine, (i.e. both pairs had intact spines and negligible pain), a list of 221 complete twin pairs (146 presumed concordant and 75 presumed discordant) born between 1961 and 1969 were identified. These pairs were then sent a detailed questionnaire inquiring about back pain, treatment for back pain, traumas to the back, and surgeries to the back. Based on answers to this survey, the first five monozygotic responders who were discordant for pain, major trauma and/or surgery and the first five monozygotic responders concordant for pain and back trauma and/or surgery, were selected and contacted by telephone to inquire about participation in the study. In case one or both twins declined to participate, the next pair on the list was approached until 5 concordant and 5 discordant pairs were enrolled in the study. All experimental procedures as well as informed consent procedures from all subjects were obtained under ethical approval from the Danish Twin Registry and the Ethics Committee for the Region of Southern Denmark approval (#S-20090112). All experiments were performed in accordance with relevant guidelines and regulations.

### Magnetic Resonance Imaging (MRI)

In all subjects, MRI was performed with the same 1.5T magnet (HDTX Twinspeed, General Electric, USA) using the same imaging protocol at the same facility. Images of each twin were obtained sequentially, on the same day, and immediately before SHM evaluation. A HNS CTL345 receiving coil was used with the participants lying in the head first supine position. A localizer sequence of eight images were obtained and consisted of four coronal, three sagittal and one transversal orthogonal images. This was followed by a sagittal T1-weighted image FSE (700/15 (TR/TE), 320 × 320 matrix, 310 mm. field of view, 15 slices of 4.0 thk/0.6 sp), a sagittal T2-weighted spin echo image (4300/108 (TR/TE), 320 × 320 matrix, 310 mm. field of view, 15 slices of 4.0 thk/0.6 sp) and an axial T2-weighted spin echo image (4300/109 (TR/TE), 288 × 256 matrix, 220 mm field of view, 42 slices of 4.0 thk/0.6 sp). Slices were localized in the plane of the five lower discs.

### SHM

**Figure 1:**
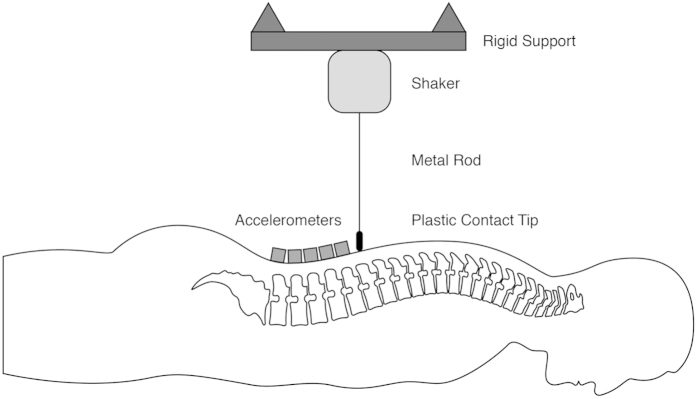
SHM equipment in a subject preparation.

SHM was performed sequentially on each twin on the same day immediately following image acquisition. Compared to previously reported SHM techniques where instruments were attached to cadaveric spines directly^16^,^17^,^18^, a non-invasive technique was developed in preliminary investigations on human cadavers and asymptomatic humans. Specifically, this non-invasive technique utilizes an electromechanical shaker (LW-126-13, Labworks, Costa Mesa, CA) supported above the prone subject (Fig. 1). A flexible metal rod extending from the shaker was used to transmit vibration to the subject by direct contact. A saddle-shaped plastic tip on the terminus of the metal rod was placed over the spinous process of twelfth thoracic (T12) vertebra with a compressive contact force of 5 N. A load cell (208C02, PCB Piezoelectronics Inc., Depew, NY) mounted in-series with the metal rod was used to measure the applied force. The applied vibration was provided in a pulse train of 10, 1-second pulses with each pulse containing randomized frequencies (1−2000 Hz). Customized software was employed to control the range of vibration frequencies and average the pulse trains (Spectral Dynamics, San Jose, CA). The excitation voltage of the shaker was held constant during all testing to limit the maximal acceleration at each frequency. By keeping the vibration amplitude constant at each frequency the system to be analyzed can be considered as linear^15^. For each pulse train, participants held their breath at full expiration and the vibration response collected by five uniaxial accelerometers (PCB Piezotronics, Depew, NY) mounted with medical-grade cyanoacrylate to the skin overlying the spinous processes of the first through fifth lumbar vertebrae (L1-L5). Spinous processes were identified by palpation performed by a trained clinician and accelerometers were placed by the same clinician. During the applied vibration pulse, acceleration data were obtained from each sensor with a computerized data collection system (Spectral Dynamics, San Jose, CA) at a collection rate of 5000 Hz as was data from the load cell. In total, three pulse trains were collected per participant with acceleration data from each sensor expressed as the ratio of the resulting frequency response spectrum in relation to the applied force (i.e. frequency response function (FRF))^20^. This approach has been shown to be reliable when sensor positions are not altered^21^.

### MRI evaluation

In each subject, MRI images were evaluated by a single board certified radiologist who was blinded to subject identity. Radiological evaluations were provided as a written report which contained specific observations for each lumbar level.

### Determination of Concordance

The MRI concordance status of enrolled twin pairs was determined by a second board certified radiologist who visualized twin MRI images together along with the written radiologic evaluations. From this process, the evaluator determined whether each spinal segment was concordant or whether one twin possessed a significant structural dissimilarity compared to the other twin (e.g. compression fracture).

### Analysis

Signal coherence was calculated as the ratio of the squared cross-spectral power and the product of the spectral powers of input (T12) and output at each sensor site. The mean and standard deviations of these values were than computed for all data at each sensor site. The frequency response function (FRF) was calculated as the ratio of the output power and input power over the frequency range applied. FRFs were smoothed using a median filter. Three different outcome measures were computed from the smoothed FRF obtained from each of the five sensors (frequency at peak power (PEAK), area under the curve (AUC) and root mean square (RMS)). AUC was calculated using trapezoidal numerical integration, while PEAK was defined as the frequency at which the FRF peak amplitude was recorded across the frequency spectrum. The difference in each outcome between twins was calculated on a per sensor basis and averaged across the three obtained trials. An analysis of covariance (ANCOVA) was then applied to determine if the mean differences of the above mentioned outcome measures were significantly different from zero (p < 0.05).

## Results

### Response rate to survey

Of the 442 twin individuals approached in the survey, 372 (84.2%) responded after one reminder yielding a total of 117 presumed concordant and 48 presumed discordant complete pairs. After careful screening of all returned questionnaires, a list of concordant and discordant twins was generated where concordance status was first based on survey results (Table 1). All 5 twin pairs on the discordant pairs list consented to participation while two concordant pairs declined participation. Consequently the next two pairs on the concordant pairs list that consented to participation were approached.

### Concordance status based on MRI

Based on MRI evaluation, 4 twin pairs were rated as concordant given their similar appearance on imaging (8 subjects in total) while 6 twin pairs (12 subjects) were rated as discordant given significant structural dissimilarities at single or multiple spinal levels (Table 1).

### Coherence results

Coherence compares input and output signals and ranges in value from 0–1 where a value of 1 indicates a linear system with no noise. In each subject, coherence was calculated from the three acquired trials and averaged for each accelerometer with the accelerometer at L1 being closest to the vibration source. The mean coherence between participants were then computed with the following results: L1 sensor (0.71 ± 0.12), L2 sensor (0.82 ± 0.09), L3 sensor (0.83 ± 0.08), L4 sensor (0.82 ± 0.09) and L5 sensor (0.15 ± .01). Based on these values, the coherence of L5 was considered to be inadequate due to sensor distance from the vibration source and data from this sensor were not considered for further analysis.

### FRF results

**Figure 2:**
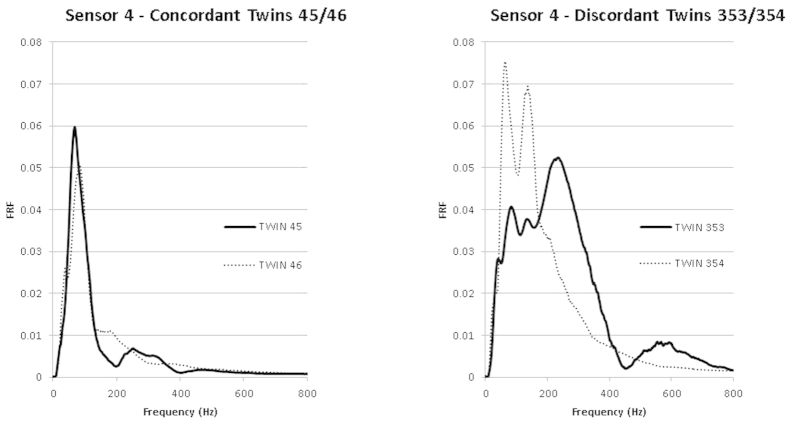
Representative FRF data from concordant and discordant twins.

Body mass index was not significantly different between twins as a group or when stratified into concordant/discordant groups (p > 0.05 in all cases). In concordant twin pairs, the three outcome measures were found to have insignificant differences between twin pairs (Table 2). In those twins judged to be discordant, two of the three outcome measures were found to have insignificant differences between twin pairs while one outcome measure (PEAK) identified significant differences in all sensors (L1-L4 inclusive, Table 2). Example FRF data for concordant and discordant twins are displayed in Fig. 2. Overall, the mean difference in the FRF between twins decreased as the sensor distance from the vibration source increased.

## Discussion

Using an identical twin study design, we have demonstrated that pathological changes within the spine alter its vibration response. In general, twins with similar spines had vibration responses that were statistically similar. Alternatively, twins with structurally dissimilar spines had vibration responses that were significantly different from each other. This study is the first to use non-invasive techniques to apply structural health monitoring principles to the spine *in vivo*.

There are several limitations related to the results of this study. First, with respect to the study design, the use of twins is an approach that effectively minimized inter-subject variation in anthropometric factors such as age, sex and body mass index. Although these variables had an insignificant impact in this study, this twin design cannot ensure that the spines of concordant twins would respond equally to vibration. Therefore, an important limitation in this study is the lack of an absolute gold-standard to which vibration response can be compared. This means that in the future, additional means of vibration response validation may be needed. Second, this study employed skin-based accelerometers that if removed (sensors were never removed during data collection), are difficult to replace in the same subject in a repeatable manner^21^. Depending on the effect size of the response, this circumstance may limit accelerometer-driven SHM from being used to monitor the spine at successive time points. In addition, differences in sensor attachment and cable placement may alter sensor response, however, if these effects were present within the study, they could be considered to be negligible given that statistically similar signals existed in concordant twins, but never in the discordant twins. Finally, it is not known how the vibration signal may be influenced as it passes through adjacent vertebrae. As such, future applications of this technique may be limited to vertebrae immediately adjacent to the site of vibration application.

As noted in the results section, the mean difference in each outcome variable decreased in magnitude as distance from the vibration source increased. The most probable explanation for this observation is soft-tissue attenuation of the vibration signal due to increased propagation distance from the source to the distant sensors as well as increased spinous process depth in the more inferior sensors (L4-L5)^22^. Further, signal coherence was significantly lower at the L5 sensor. This lack of coherence, in addition to a decrease in signal magnitude, suggests that at some point, the distance of the sensor from the vibration source may be too great with the result that the signal to noise ratio becomes insufficient for meaningful data analysis.

Given the established use of SHM in evaluating industrial structures and the successful adaptation of SHM techniques for assessing cadaveric spines^16^,^17^,^18^, assessing the performance of SHM principles *in vivo* is a logical next step but one facing significant challenges.

First, many aspects of vibration testing require a linear system to be valid. While biological tissues are non-linear, there are circumstances when these non-linear effects can be minimized, as was the case in this experiment. Specifically, we do not attempt to assess vibration responses over a range of vibration magnitudes – each frequency applied to the spine reamins at the same magnitude within the same testing session. We also see that signal coherence in the majority of sensors is of adequate magnitude, which suggests that vibration transmission is sufficient and attenuated minimally through soft tissues. In addition, vibration is applied to a single site without the subject changing positions or any other circumstances that would require the vibration contact or sensors to be replaced. Taken together, these features allow us to obtain a “linear equivalent” where the system response is relatively constant.

Second, three SHM outcomes were assessed for their ability to discriminate between concordant and discordant twins. In all cases, these outcome measures were single values used to represent two dimensional FRFs. Only one of these outcome measures (PEAK) was found to be responsive. We speculate that the responsive nature of PEAK as an FRF outcome measure is in part due to large magnitude changes in FRF amplitude response associated with the gross structural alterations in discordant twins. Therefore, should some as yet unidentified structural alterations, pathologies or injuries of the spine be characterized by small changes in FRF, they may not be identified by this SHM using PEAK.

Interestingly, the structural alterations represented here were visually unique between discordant twins (Fig. 2). This observation suggests that various spinal conditions, pathologies and injuries may have unique FRF “fingerprints”; a circumstance observed using invasive SHM techniques in cadavers^17^. Although the presence of unique FRF fingerprints for specific pathologies cannot be queried by our study design, our data suggests that further investigation into the application of SHM in large human populations is justified in that SHM could provide a diagnostic alternative to existing procedures that are difficult to access, invasive or costly.

## Conclusion

The present study demonstrates that structural changes within the spine alter its vibration response significantly.

## Additional Information

**How to cite this article**: Kawchuk, G. N. *et al.* Structural health monitoring (vibration) as a tool for identifying structural alterations of the lumbar spine: a twin control study. *Sci. Rep.* **6**, 22974; doi: 10.1038/srep22974 (2016).

## Figures and Tables

**Table 1:** Concordance status based on self-reported symptoms and MRI.

Status	Concordance by Self-Report	Twin#	Sex	Age	BMI	Concordance by MRI	Status
Some LBP in past year	Concordant	19	F	48	29.39	Concordant	Normal
Some LBP in past year	Concordant	20	F	48	31.23	Concordant	Normal
No history of LBP	Concordant	45	M	47	27.12	Concordant	Normal
No history of LBP	Concordant	46	M	47	24.82	Concordant	Normal
No history of LBP	Concordant	85	M	46	28.71	Concordant	Normal
No history of LBP	Concordant	86	M	46	24.97	Concordant	Normal
No history of LBP	Concordant	125	F	43	22.13	Discordant	Normal
No history of LBP	Concordant	126	F	43	23.3	Discordant	Hemangioma L4
3 days mild LBP in last year	Concordant	189	M	48	22.13	Concordant	Normal
3 days mild LBP in last year	Concordant	190	M	48	23.3	Concordant	Normal
No history of LBP	Discordant	13	M	44	20.58	Discordant	Normal
Compression fracture 1996	Discordant	14	M	44	21.45	Discordant	Comp.Fracture L1
Daily LBP with sciatica.	Discordant	299	F	48	27.1	Discordant	Disc Degeneration L1
No history of LBP	Discordant	300	F	48	25.59	Discordant	Normal
No history of LBP	Discordant	353	M	45	23.55	Discordant	Schmorl’s Node L2
Daily LBP/Prior disc surgery	Discordant	354	M	45	26.3	Discordant	Laminectomy L4-5
No history of LBP	Discordant	391	M	43	31.46	Discordant	Hemilaminectomy L4
Prior disc surgery	Discordant	392	M	43	31.86	Discordant	Disc Degeneration L3-4
Frequent LBP	Discordant	393	F	43	26.03	Discordant	Normal
No history of LBP	Discordant	394	F	43	26.13	Discordant	Disc Prolapse L4-5

**Table 2:**
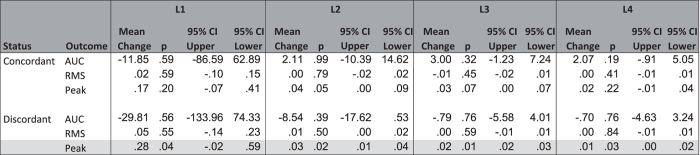
Mean observed difference values and p-values from paired t-test analysis between identical twins within a twin pair.

Data are presented for three outcome measures (area under curve, AUC; root mean square, RMS; peak frequency, Peak). Grey shading indicates statistically significant differences (p < 0.05) between the mean difference and a hypothesized difference of zero.
